# Cannabis use during pregnancy and hemodynamic responses to infant cues in pregnancy: an exploratory study

**DOI:** 10.3389/fpsyt.2023.1180947

**Published:** 2023-09-06

**Authors:** Shannon Powers, Xu Han, Jacqueline Martinez, Alexander John Dufford, Torri D. Metz, Tom Yeh, Pilyoung Kim

**Affiliations:** ^1^University of Denver, Psychology, Denver, CO, United States; ^2^University of Colorado, Computer Science, Boulder, CO, United States; ^3^Department of Medical Social Sciences and Institute for Innovations in Developmental Sciences, Northwestern University Feinberg School of Medicine, Chicago, IL, United States; ^4^University of Utah Health, Obstetrics/Gynecology, Salt Lake City, UT, United States; ^5^Department of Psychology, Ewha Womans University, Seoul, South Korea

**Keywords:** cannabis, pregnancy, fNIRS (functional near infrared spectroscopy), parenting, psychology

## Abstract

**Introduction:**

Cannabis is one of the most commonly used substances during pregnancy and has the potential to negatively impact parent-infant relationships. The prefrontal cortex (PFC) response to infant cues during pregnancy has been associated with subsequent positive parenting behaviors. However, PFC activation is altered in individuals who use cannabis. As the potency of cannabis has changed over the years, little is known about the specific role of cannabis use on gestational parent brain responses to infant cues.

**Materials and methods:**

Using functional Near-Infrared Spectroscopy (fNIRS) in the second trimester of pregnancy, we measured hemodynamic responses to an infant cry task and an infant faces task among individuals who were using cannabis (*N* = 14) and compared them with those who were not using cannabis (*N* = 45). For the infant cry task, pregnant individuals listened to cry sounds and matched white noise. For the infant faces task, they viewed happy, sad, and neutral faces.

**Results:**

There was no significant difference between the two groups after adjusting for multiple comparisons. Without adjusting for multiple comparisons, we found preliminary evidence for the differences in the dorsomedial PFC associated with heightened response to infant cry among individuals who use cannabis. The groups were also different in the dorsolateral PFC associated with decreased response to infant sad faces among individuals who use cannabis.

**Discussion:**

Our preliminary data suggests that cannabis use during pregnancy was associated with brain activation in the regions involved in the emotional regulation and information processes. However, the results did not survive after adjustment for multiple comparisons, thus future research with larger sample sizes is needed to confirm potential differences in brain function among cannabis-using pregnant individuals.

## Introduction

Cannabis is the most commonly used substance during pregnancy, with prevalence of use estimates ranging from 5–20% among pregnant individuals in the United States (1–4). In a report from the Colorado State Pregnancy Risk Assessment Monitoring System, 8.2% of people reported using cannabis during pregnancy from 2014–2018, a higher rate than cigarette and electronic cigarette use in Colorado (4). With increasing legalization of cannabis across the United States, it is imperative to further investigate the implications of cannabis use during pregnancy.

Pregnancy is a sensitive window for expecting birthing parents to develop neural and emotional bonds with the fetus. The structural and functional brain changes that occur during pregnancy translates to early infant bonding (5–9). During the initial postpartum period there are increases in grey matter volume in sections of the prefrontal cortex (10–14). Another study looking at the long-term effects of pregnancy found reductions in grey matter in areas revolved around social cognition including the superior temporal sulcus, medial and inferior frontal cortex, and bilateral prefrontal cortex associated with enhanced parent–child attachment (6). In a recent review combing both animal and human literature, Duarte-Guterman found overall decreases in volume, neurogenesis, dendritic morphology, and number of microglia; and increases in dendritic spine density in the hippocampus and prefrontal cortex during the postpartum period due to pregnancy (15). Newer research in animal models has shown that many of these modifications during pregnancy occur during the gestation and lactation period and that these changes predict maternal caregiving behaviors postpartum (16). The vast complexity of these structural changes shows the wide network arrangements that begins during pregnancy to prepare mothers for their infant and the importance of the prefrontal cortex in these changes.

In addition to structural changes in the gestational parent brain, studies focused on parental brain function suggest that neural responses to infant cues during pregnancy are important to support more positive parent-infant relationships postpartum (5, 17). Infant cues such as cry sounds or emotional facial expressions are important not only for communication, but also promote care between a parent and child (18). This sensitivity to infant cues within parental reward and motivation networks is thought to be related to increases in oxytocin and dopamine networks during late pregnancy and the first months of the postpartum period that are associated with birthing parent behavior (19–23). Activation of brain networks for both looking at your infant, and responding to cries involve several brain regions including the prefrontal cortex regions (24–26). Literature has attempted to assess differences of emotional valence of infant faces, and found variability in responses can be predictive of parenting behavior postpartum (27, 28). Studies have shown that own smiling, compared to other smiling infant faces elicit social information and reward/motivation regions of the cerebrum, midbrain, orbitofrontal cortex tracks, including substantia nigra and amygdala (12, 29–31) When comparing nulliparous individuals to birthing parents, nulliparous individuals show slight activation in regions of the medial prefrontal cortex and posterior cingulate cortex in the context of goal oriented tasks, highlighting a potential sensitivity among birthing parents (32).The medial PFC regions are important for perception and evaluating the meaning of the infant’s emotional cues while the lateral PFC regions are important for regulating a birthing parents’ own emotional reactions (24, 26, 33–35).

Disruptions to these functional changes in the PFC during pregnancy to help gestational parents regulate emotions, such as depression and substance use, has been associated with disruptions in parent-infant attachment (36). While often there is an increase in activation toward infant cues in parenting neural networks associated with positive behavioral outcomes, contrarily heightened activation in the PFC can be associated with dysregulated responses. In a review on plasticity during pregnancy, it was found that stress exposure—in the form of childhood maltreatment, environmental stress, and parenting stress—can cause dysregulated levels of activation in the PFC in response to infant cues in regions of gestational parent motivation, emotion regulation and empathy (37). Both heightened and dampened responses in parental networks from stress exposure impacted neural responses and behavioral outcomes, highlighting the complex nature of an appropriate attenuated response to infant cues.

Cannabis exposure during pregnancy has been difficult to characterize due to decrease and variability of exposure as pregnancy progresses with the highest prevalence during the first trimester, and dropping by half in the third trimester (38–43). Current literature states that many pregnant individuals report cannabis use and perceive that cannabis use is safe (43–45). Limited research on prenatal cannabis exposure focuses on infant outcomes. Cannabis exposure during pregnancy is associated with decreased birth weight, reduced length of infant, smaller head circumference, and higher risk of preterm birth (38, 39, 46). Since human data is limited, animal models have shown that cannabis exposure during pregnancy is associated with diminished pup rearing behaviors in open field (47, 48). Cannabis use Disorder, known as CUD, has been shown to be associated with lower positive parenting through decreased monitoring, support and consistency in adolescents (48). Gestational parents who co-used tobacco and cannabis during pregnancy showed lower sensitive parenting behaviors with their infants at 9 months (47). Previous studies have also examined the effects of polysubstance use on postpartum parental brain function. Postpartum participants who used substances exhibited a decrease in activation in the hypothalamus, ventral striatum, and ventral lateral PFC to happy infant faces compared with healthy controls (11, 12, 17, 49). When investigating own versus other infant images, gestational parents who use substances showed reduced activation to happy and sad faces in their own infants compared with individuals who do not use substances in regions of the dorsolateral PFC and ventral medial PFC (50). When investigating infant cry, some research has found no difference between those who do and do not use substances, while others have found overall reduced activation in the PFC (27, 50). However, to date, there is limited evidence focusing on cannabis use (38). Based on the variability of cannabis exposure during pregnancy and conflicting findings among substance exposure, this study encompassed any individual who self-reported or had a positive toxicology report during the second trimester.

Functional Near-Infrared Spectroscopy (fNIRS) and EEG studies are the primary resources for evaluating changes in the brain during pregnancy. Recently, fNIRS has increased in use and provided the tools to measure hemodynamic responses during pregnancy, a technique more comparable to MRI and has better spatial resolution than EEG (51–53). Two key advantages of fNIRS are the ability to account for motion, and the ability to measure during pregnancy, a time where MRI is more limited in its use (52, 54). Previous research using fNIRS has documented changes related to infant stimuli during pregnancy (52, 54). Length and bias of attention to infant cues assessed in the third trimester is associated with sensitivity postpartum (55). However, PFC activation measured by fNIRS was most pronounced in the second trimester, compared to other trimesters in response to emotional cues (i.e., adult fearful faces) (52). Thus, the brain responses to infant cues earlier during pregnancy, such as in the second trimester, may also be important for predicting postpartum brain function. With the advantages of fNIRS, there are limitations to its use as fNIRS can only measure the hemodynamic response of cortical surface activity.

This study examined the associations between cannabis use and hemodynamic responses to infant cues measured by fNIRS in the second trimester. We hypothesized that there would be less hemodynamic activation in regions of the PFC to infant faces, in particular to happy and sad faces based on the study investigating substance exposure in PFC regions, and a reduction of activation to infant cry in regions of the PFC measured by the hemodynamic response as fNIRS can only measure cortical surface activity (27, 50).

## Materials and methods

### Participants

Participants were pregnant individuals recruited up to 14 weeks’ gestation from the Department of Obstetrics and Gynecology at Denver Health medical center, through the University of Colorado Anschutz Medical Campus, and through flyers and brochures as part of a larger study investigating the effects of income during the pregnancy period. A subset of this study investigated the effects of cannabis exposure during this time-period. Eligibility criteria included: age 18–45 years, singleton pregnancy, and fluency in English. Exclusion criteria included: use of current psychotropic medications; lifetime diagnosis of other psychiatric/neurological illness other than depression, anxiety, or post-traumatic stress disorder; positive urine drug screen for non-cannabis illicit substances; or self-reported heavy nicotine or alcohol use. Participants for these analyses were grouped as using or not using cannabis based on a second trimester urine toxicology test for cannabis or self-report as cannabis use during this time has been shown to be more sporadic among pregnant individuals. Participants completed mood measures, income interviews, urine toxicology and self-report cannabis exposure, and fNIRS tasks in their home. Participants were compensated 75$ for the visit.

Of the 74 participants that had completed fNIRS imaging prior to March 5, 2020, 14 participants tested positive for or reported cannabis use in the second trimester and were assigned to the cannabis group. For the comparison group, there were 60 participants eligible for analysis who had completed fNIRS imaging. Of these 60 eligible participants for the control group, 15 were excluded for the following reasons: (*n* = 10) reported cannabis use from conception to their first trimester visit or tested positive at their first trimester visit but did not continue use in the second trimester, (*n* = 4) missing yearly income data, and (*n* = 1) for completing the second trimester visit in their third trimester. The demographic breakdown of each group is listed in Table 1.

**Table 1:** Characteristics of participants.

	Control *n* = 45	Cannabis exposed *n* = 14	
	Mean +/− Standard deviation (Range)	Mean +/− Standard deviation	*p* value^*^
Nulliparous	11	7	–
Gestation Weeks	23.20 +/− 1.59 (20–28)	23.29 +/− 1.73 (21–28)	–
Education (years)	15.27 +/− 2.615 (12–20)	13.64 +/− 2.31 (12–20)	0.042
Age at 1^st^ Trimester	30.31 +/− 4.71 (18–41)	28.43 +/− 6.16 (21–37)	–
CESD	11.60 +/− 7.58 (1–33)	13.93 +/− 5.12 (3–24)	–
STAI	28.40 +/− 7.65 (20–48)	28.50 +/− 6.27 (22–46)	–
EPDS	5.07 +/− 4.63 (0–18)	6.07 +/− 3.10 (0–13)	–
Hispanic	14	2	–
Race			<0.001
American indian	2	0	
Asian	2	0	
Black	3	9	
White	29	4	
Other	9	1	
Cannabis drug screen			
1^st^ trimester drug screen positive	0	10	<0.001
2nd trimester drug screen positiveᵒ	0	8	<0.001
TLFB			
Pre-conception units	0	297.93 +/− 789.09 (0–3005.00)	–
1^st^ Trimester units	0	190.29 +/− 486.78 (0–1840.00)	–
2^nd^ Trimester units	0	231.43 +/− 501.97 (0–1820.00)	–
Tobacco use at 1^st^ Trimester	3	4	0.027
Medicare	19	11	0.018
Total yearly income at 1^st^ Trimester	88,890.76 +/− 75,565.38 (1,500–504,000.00)	42,806.07 +/− 34,669.67 (4,000–140,000.00)	0.032

*p values for less than 0.05 are shown when groups are significantly different form each other; ᵒMissing drugscreen at second trimester visit (control *n* = 22; cannabis exposed *n* = 4); CESD is the Center for Epidemiological Studies-Depression; STATE is the State–Trait Anxiety Inventory-state; EPDS is the Edinburg Postnatal Depression Scale; TLFB is the Timeline Follow Back Scale.

### Procedures

Participants were initially contacted by phone or in the prenatal clinic at Denver Health Hospital (Denver, Colorado) to assess their eligibility for the study. If they were eligible, a home visit was conducted during each trimester. During the first and second home visit, participants were consented and completed measures of mood, income, demographics, a urine drug screen, timeline follow back data, and the fNIRS tasks. fNIRS tasks included infant cry, infant faces, and adult faces tasks. Only data from the infant cry and infant picture tasks will be examined in this paper.

### Measurements

#### Cannabis use

1. *Urine Testing/Nicotine Testing.* After consent, participants provided a urine sample for a Nic-Alert test to evaluate for nicotine metabolite and a CLIA-waived 5-panel drug immunoassay at their first trimester study visit. If participants tested positive for any other substances besides cannabis and nicotine, they were ineligible for the study. Participants who tested positive for cannabis were used for the present analysis. Because urine drug testing at the second trimester study visit was added later in the study, not all participants have a urine drug screen for this visit (*n* = 33 total missing tests in the second trimester).
2. *Timeline Followback/Self-Report Drug Use Scale (TLFB).* Since cannabis use can be infrequent, and urine can be negative despite recent use, all participants also underwent a detailed interview by a trained researcher who asked about any cannabis use prior to the visit. This interview methodology has been shown to be an accurate reflection of self-reported cannabis use (56–58). Participants who self-reported cannabis in the second trimester but did not complete the TLFB during their second trimester were also put into the cannabis group.

### Mood measures

Participants completed three mood measures: Edinburg Postnatal Depression Scale (CESD), and State–Trait Anxiety Inventory (STAI)-state. Participants completed the EPDS asking about their feelings in the past 7 days (59–61). Participants completed the CESD, a 20-item questionnaire asking them about how often in the past week they experienced symptoms associated with depression (62–64). Participants completed the STAI, a 20-item questionnaire about how they are feeling right now at this moment (65, 66).

### fNIRS (functional near-infrared spectroscopy)

Choosing a region of interest approach, we used fNIRS Optodes’ Location Decider (fOLD) to choose a prefrontal montage that would highlight the dorsolateral PFC and dorsomedial PFC (67, 68) (see Supplementary Figure S1 for regions covered by a prefrontal montage). Two key advantages of fNIRS are the ability to account for motion, and the ability to measure during pregnancy (51–53). The infant cry and infant face paradigms have been evaluated in research to show enhancement of hemodynamic activation associated with parenting across multiple studies (8, 10, 11, 36). In addition, these tasks have been validated among individuals with substance use disorders (27, 49, 50).

#### Infant cry paradigm

All participants listened to the same infant cry sound and matched white noise to that cry using Cool Edit Pro 2.0 sound editing software (69). Samples of natural infant cries in a previous project in the lab were collected during a diaper change and validated for average emotional intensity (70). All participants listened to the same infant cry sound. Participants listened to the task through headphones all on the same volume of 22. The paradigm was organized in E-Prime 2.0 by two 10 s blocks, cry and matched white noise, with a 10-s rest with a crosshair design of silence between each sound (71). In each block there were 10 cry sounds, and 10 matched noise sounds randomly presented to the participants. Overall, there was a total of 20 trials lasting 8.3 min. Participants were asked to listen and pay attention to the sounds and let themselves experience the thoughts and feelings they were having naturally. After each sound block, they are asked to rate each sound on a Likert scale from 1-very negative, 3-neutral, to 5-very positive.

#### Infant faces paradigm

Participants were given a task during which they viewed either White or Black infant faces matched to the race of the expected child. For races other than Black or White, participants viewed White infant faces. There were male and female infant faces (12). The paradigm was organized in E-Prime 2.0 by three 8-s blocks: happy faces, neutral faces, and sad faces (71). In each block, 4 faces of the same emotion were shown for 2 s each, for a total of 8 s. In between the blocks there was a 10 s rest with a crosshair design. Overall, there was a total of 30 trials lasting 9.3 min. Participants were asked to pay attention to the images and let themselves experience the thoughts and feelings they were having naturally.

### Data acquisition and processing

Cortical activation was measured using the NIRx NIRSCOUT fNIRS System. Using the prefrontal montage designed by NIRx consisting of 15 optodes (8 sources and 7 detectors) with an average 3 cm distance between each optode were placed over the PFC using a NIRSCap for a total of 20 predetermined channels (see Supplementary Figure S1). The NIRS method uses infrared light sent through fiber optic cables of the 8 source optodes using 760 nm to measure oxygenated hemoglobin (HbO) and 850 nm for deoxygenated (HbR) hemoglobin. Light passes through the cortical layer of the cortex and the reflection to the detectors represents the change in oxygenated and deoxygenated hemoglobin concentrations using Beer’s Lambert Law (72, 73). At the start of the paradigm, participants’ caps were placed one inch above the eyebrow and a calibration check was performed to confirm probes were getting optimal signal. Once calibrated, and all possible additional light was blocked form the probes using a blacked-out shower cap, participants began the fNIRS paradigm seated at a table with a laptop in front of them. Light was emitted continuously through the tasks.

### Analysis

fNIRS data analysis was conducted through MATLAB 2019b using NIRS Toolbox version 837 and SPSS using all 20 channels (74–76). Data were first pre-processed on the individual level. Data were quality checked, resampled from 10 Hz to 4 Hz, and deoxygenated and oxygenated hemoglobin was converted using a modified Beer–Lambert Law (77). Signal preprocessing was completed using a PCA filter. Next, a general linear model was run on the first level of analysis using AR-IRLS, this model has been previously validated and was created with the NIRx team (75). The beta values of each condition (infant cry and white noise; happy, sad, and neutral) from the first-level analysis were extracted and second level analysis was completed in SPSS. Beta values 3 standard deviations above the mean were identified as outliers and excluded from analysis (78, 79). This paper will present oxygenated results; for deoxygenated results, please see Supplementary material.

Overall averages of behavioral responses were compared between groups using a repeated measures ANOVA. Group differences for demographic variables were also tested. For the hemodynamic data, a repeated measures ANOVA model was run between groups (cannabis use and non-use), while covarying for self-reported yearly income in SPSS. Identified channels were used in an independent means *t*-test to identify directionality between conditions.

The current study included a relatively small sample of the cannabis exposed pregnant individuals due to the difficulties in recruitment. The goal of the study is to provide pilot findings for future studies with a larger sample size. Thus, the results were reported without adjusting for multiple comparisons. However, for completeness of the analysis, Benjamini-Hochberg test was performed on the significant channels that reached the threshold after adjusting for multiple comparisons.

## Results

### Characteristics of participants

Overall, 59 pregnant participants were included. There were no between-group differences in weeks of gestation, parity, or mood measures (see Table 1). There were between group differences in education level (*t* (57) = 2.083, *p* = 0.042) and yearly income (*t* (57) = 2.20, *p* = 0.032), with the cannabis exposed group having lower overall education and income as measured at the first trimester. As education and income were correlated (*r* (59) = 0.262, *p* = 0.049), only income was selected as a covariate due to a more highly significant difference between groups. There was no association between cannabis use frequency and mood measures. Participants were asked to report on a scale of 1–5, “How the sound made you feel?” with 1 being Very Negative, 2 Negative, 3 Neutral, 4 Positive, and 5 Very Positive. All but one participant in the control group made behavioral responses, and all participants in the cannabis exposed group made behavioral responses. There was a trending difference between ratings for the infant cry or matched white noise between groups (*F* (1,56) = 3.692, *p* = 0.06). The cannabis group reported the infant cry sound less negative (more neutral, 2.83+/−1.06) compared to the control group (2.14 +/−0.71), *t* (56) = −2.78, *p* = 0.007. There were no differences in responses to white nise between groups, *t* (56) = −0.72, n.s.

### fNIRS analysis of the association between cannabis use and brain activation to infant cry

**Figure 1:**
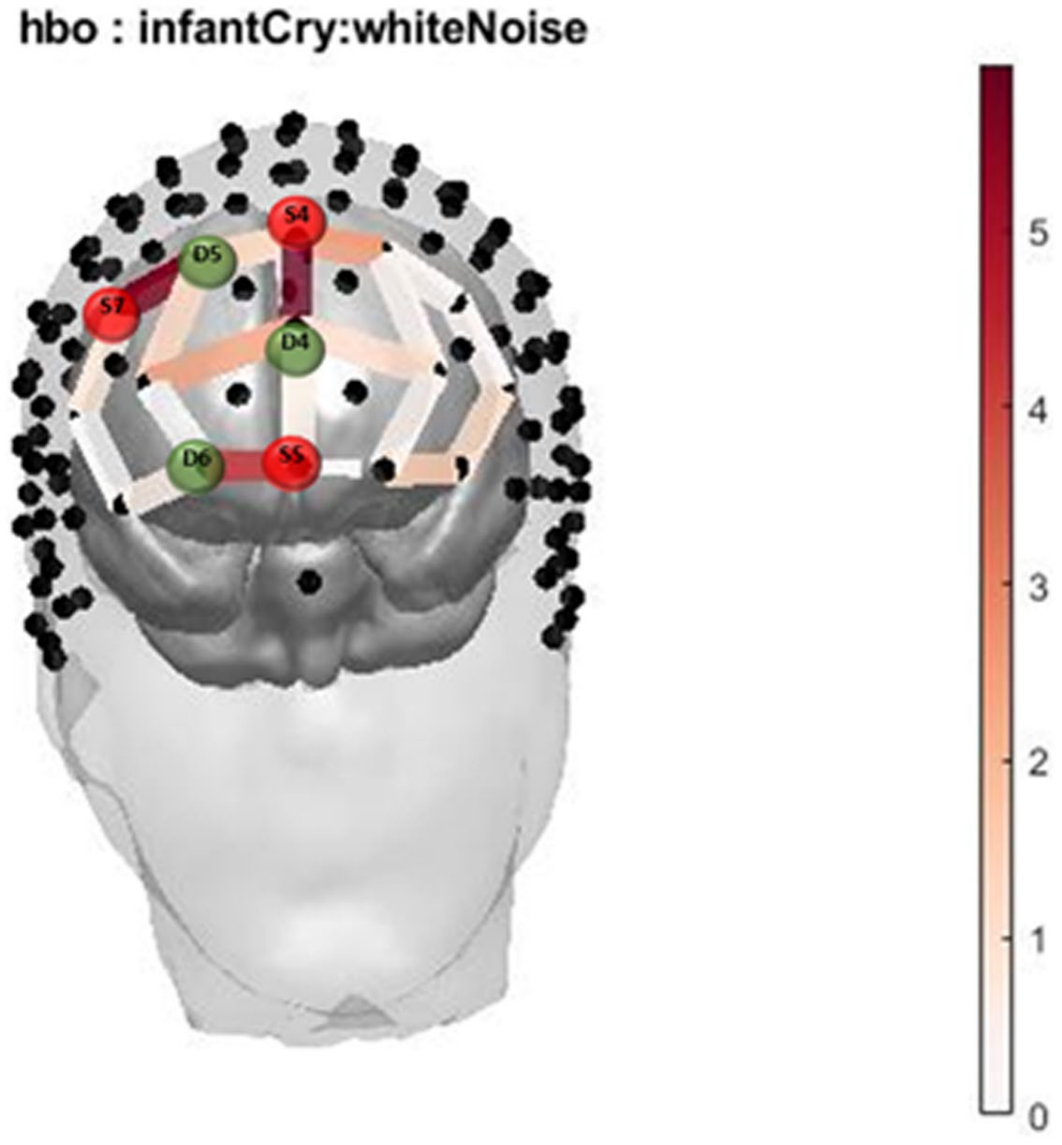
Infant Cry HbO activation. Oxygenated hemoglobin (HbO) levels of activation measured by F state size between groups in relation to location on the prefrontal montage for infant cry and white noise.

**Figure 2:**
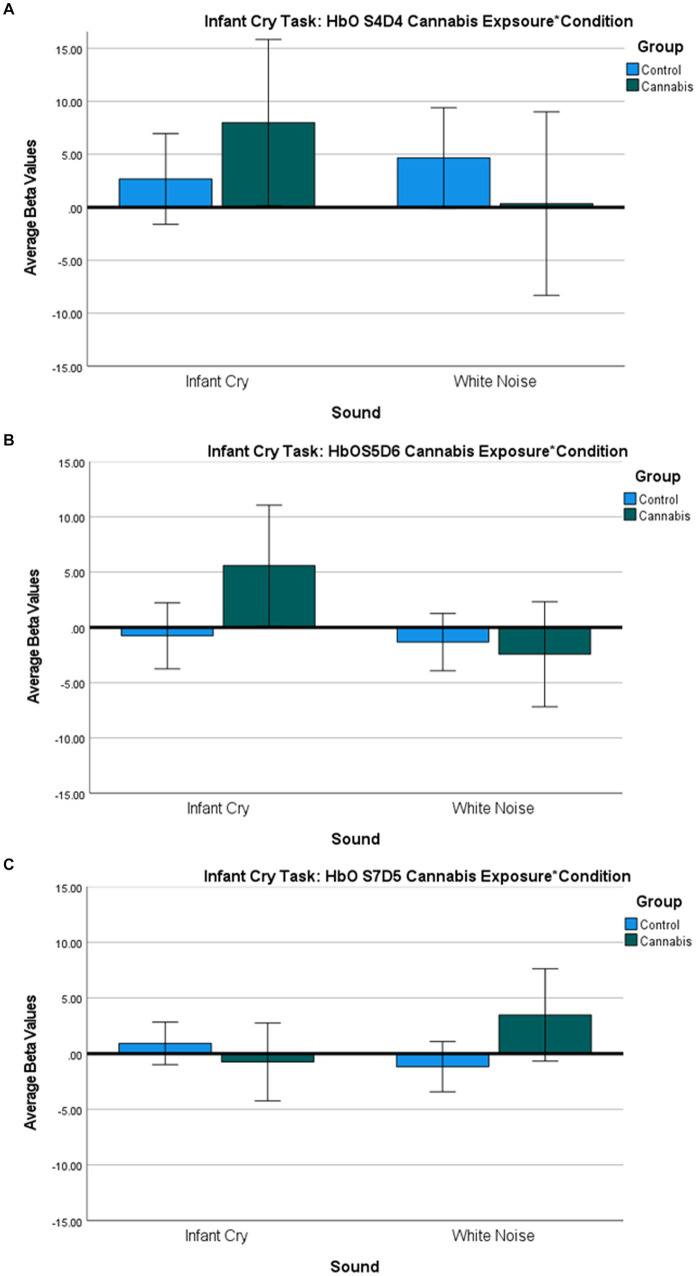
**(A-C)**: Infant Cry HbO Activation Group*Condition covarying for income ANOVA Bar Graph Results. **(A)** Average Oxygenated hemoglobin (HbO) levels between groups for infant cry and white noise for S4D4, regions of the dorsomedial PFC. **(B)** Average Oxygenated hemoglobin (HbO) levels between groups for infant cry and white noise for S5D6, regions of the dorsomedial PFC. **(C)**: Average Oxygenated hemoglobin (HbO) levels between groups for infant cry and white noise for S7D5, regions of the dorsolateral PFC.

The two-way interaction between sound (infant cry and matched white noise) x group (with and without cannabis use) covarying for income at their first trimester visit was found across 3 oxygenated channels in the right and middle dorsomedial PFC and right dorsolateral PFC (Table 2; Figures 1, 2A–C). As shown in Figure 1, in the dorsomedial PFC channels, individuals in the cannabis group exhibited increased activation to the infant cry compared with the control group (channels 36 and 47), and increased activation to white noise compared with the control group in the dorsolateral PFC (channel 34). However, the results did not survive after adjusting for multiple comparisons.

**Table 2:** Infant cry task group*Condition covarying for income ANOVA Results.

Source: Detector	Channel	Region	BA	Hemoglobin	F	*p* value
4:4	36	DMPFC	9	HbO	5.935	0.018
5:6	47	DMPFC	10, 11	HbO	4.219	0.045
7:5	34	DLPFC	46	HbO	5.573	0.022

*p* < 0.05, uncorrected; BA, Broadman area; HbO, oxygenated hemoglobin, DLPFC, dorsolateral prefrontal cortex; DMPFC, dorsolateral prefrontal cortex.

### fNIRS analysis of the association between cannabis use and brain activation to infant faces

**Figure 3:**
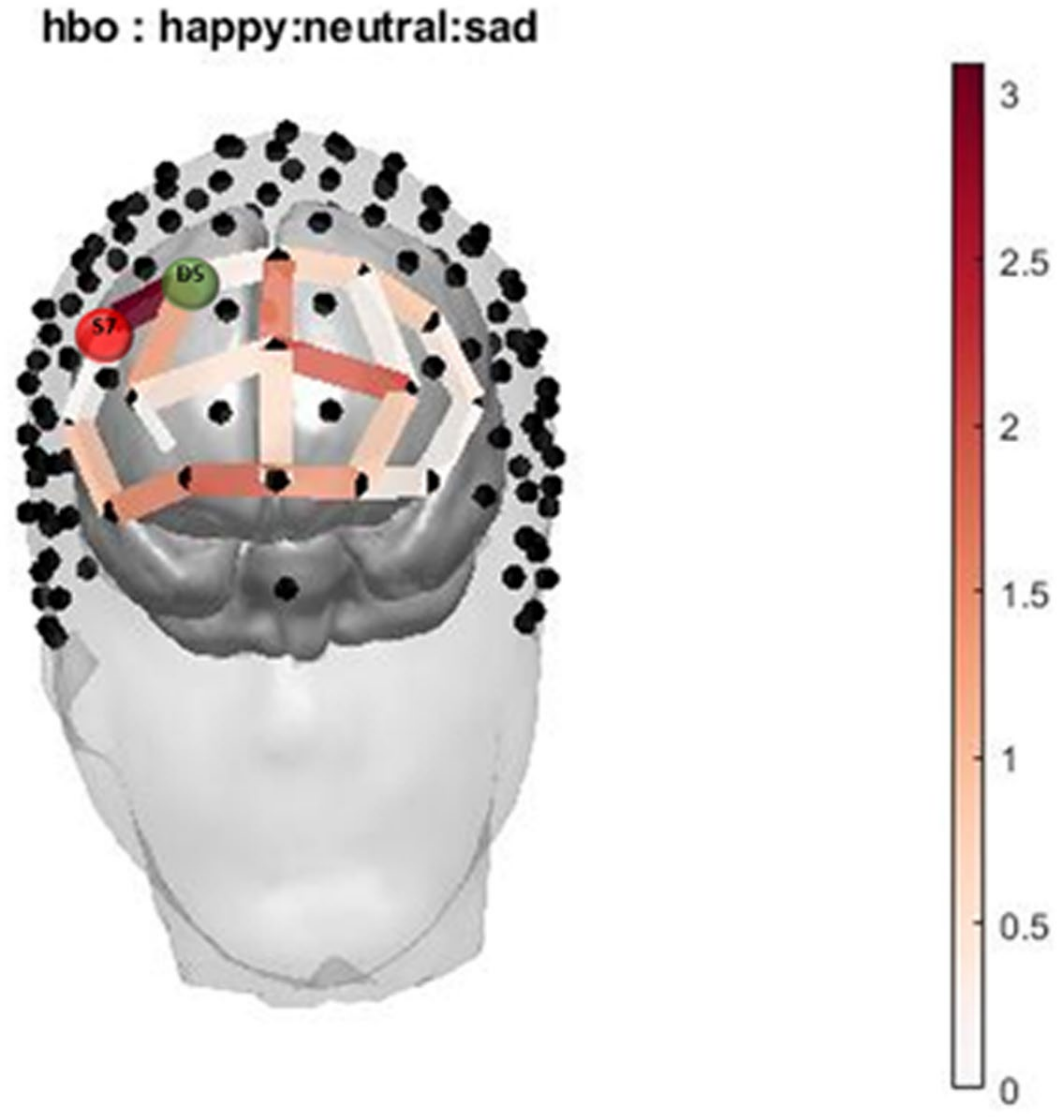
Infant Picture HbO activation. Oxygenated hemoglobin (HbO) levels of activation measured by F state size between groups in relation to location on the prefrontal montage for happy, neutral, and sad faces.

**Figure 4:**
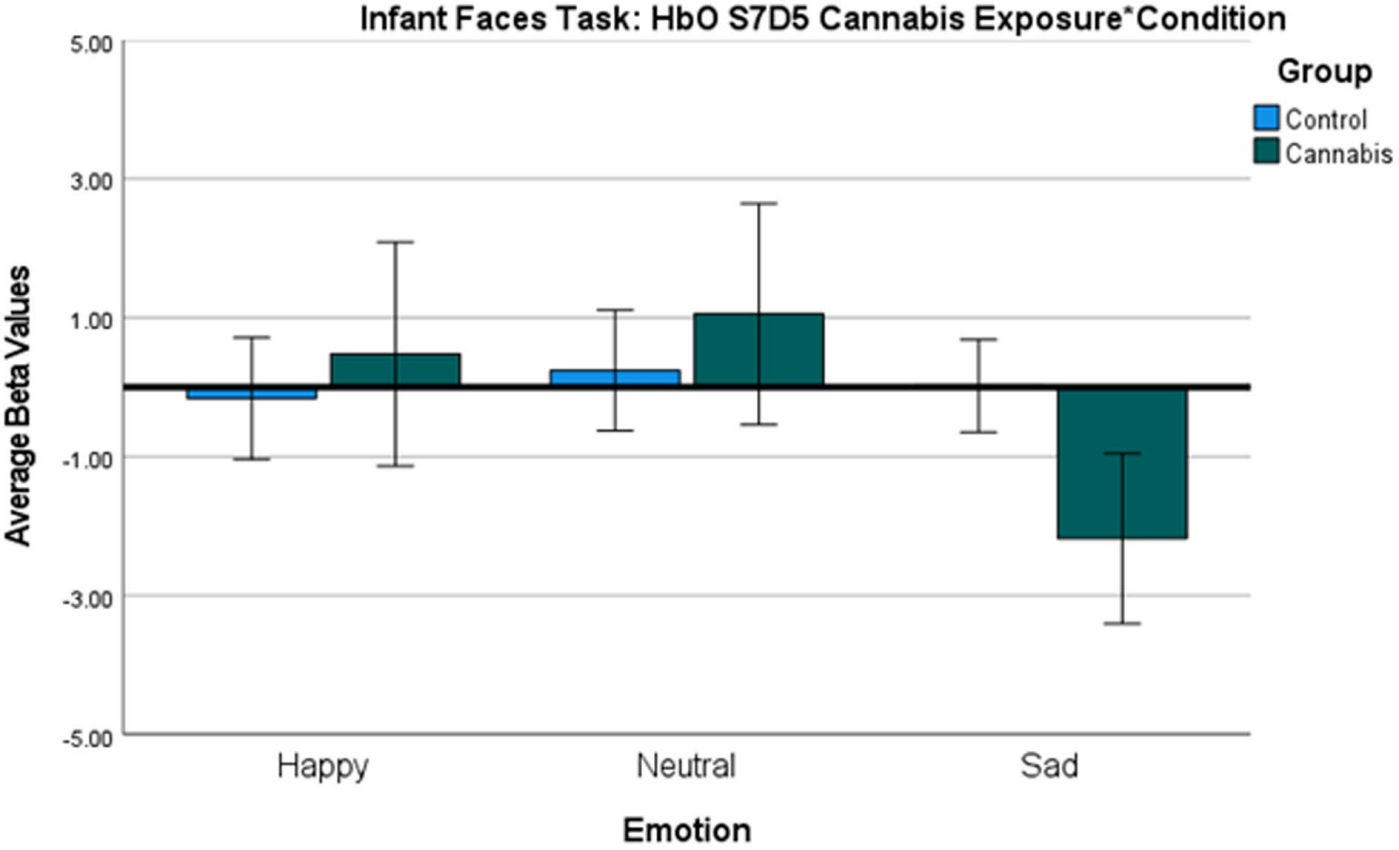
Infant Face HbO Activation Group*Condition covarying for income ANOVA Bar Graph Results. Average Oxygenated hemoglobin (HbO) levels between groups for happy, neutral, and sad infant faces for S7D5, regions of the dorsolateral PFC.

The two-way interaction between emotion (happy, sad, and neutral) x group (with and without cannabis use) covarying for income was found across 1 oxygenated channel in the dorsolateral PFC (Table 3; Figures 3, 4). As shown in Figure 3, in the dorsolateral PFC channel, individuals in the cannabis group exhibited deactivation to sad faces compared with the control group (channel 34). However, the results did not survive after adjusting for multiple comparisons.

**Table 3:** Infant picture group*Condition covarying for income ANOVA Results.

Source: Detector	Channel	Region	BA	Hemoglobin	*F*	*p* value
7:5	34	DLPFC	46	HbO	3.092	0.049

*p* < 0.05, uncorrected; BA, Broadman area; HbO, oxygenated hemoglobin; DLPFC, dorsolateral prefrontal cortex.

## Discussion

Understanding potential implications for cannabis use during pregnancy is important to help inform pregnant individuals about risks and enable them to make informed choices about use. Our study aimed to provide preliminary evidence for an association between cannabis exposure during pregnancy, and differential hemodynamic responses to infant cues. Cannabis exposure is associated with an increase response in regions of the dorsomedial PFC, and a decrease response in the dorsolateral PFC in response to infant cry. In conjunction with previous literature, we found decreased response to sad faces among individuals exposed to cannabis (27, 49). While our preliminary results were not significant after multiple comparisons correction, these data are hypothesis-generating and are intended to help inform future studies with a larger cannabis exposed sample size.

In the infant cry task, individuals in the cannabis group exhibited increased brain responses in the dorsomedial PFC (Channels 36 and 47) to the infant cry sounds vs. white noise sound compared with the control group. This finding contradicts previous literature on dampened or no response to infant cry in the postpartum period (27, 50). The observed increase in response among individuals who use cannabis may reflect more efforts for emotional information processing based on trending reports of rating the cries more neutrally. On the other hand, in the dorsolateral PFC (Channel 34) we saw increased activation to white noise in the dorsolateral PFC in oxygenated hemoglobin. Increased activation to white noise in the dorsolateral PFC may suggest that cannabis use is associated with heightened brain sensitivity to negative auditory cues. It will be important for future research to investigate how increased brain sensitivity to white noise in the dorsolateral PFC and heightened brain response to the infant cry in the dorsomedial PFC among pregnant individuals who use cannabis might be associated with later changes in parenting behaviors.

In the infant faces task, individuals in the cannabis group exhibited decreased brain response in the dorsolateral PFC (Channel 34) to sad faces compared with the control group. These findings are similar to postpartum findings for decreased response to sad faces in those with substance use disorder (27, 49). Decreased activation to sad faces among individuals who use cannabis could be related to less effective regulation of emotional responses (27, 49, 80).

This study had several limitations. Due to the nature of cannabis exposure during pregnancy, a simple dichotomous variable was used for cannabis so we were unable to examine whether the amount of cannabis affects our outcomes. In addition, some participants did not have a urine drug test at their second visit (n = 26). Future studies should use serial biospecimens to estimate total cannabis exposure across pregnancy.

A longitudinal study is needed to examine the effects of cannabis use on brain responses to infant cues during the third trimester of pregnancy and the postpartum period. In addition, a longitudinal study could help to determine whether the differences in neural response to infant cues during pregnancy associated with cannabis use led to future differences in parent–child interactions. Future studies should also investigate participant self-reported effects of cannabis safety and efficacy.

This study provides preliminary evidence demonstrating an association between cannabis use and hemodynamic response to infant stimuli. Appropriate hemodynamic activation in the dorsomedial PFC, as well as the dorsolateral PFC is necessary during pregnancy to help prepare the pregnant individual to attend to infant stimuli, but also important for understanding infant cues (19–23). However, it is important to note that the result of this study were exploratory and did not survive the corrections for multiple comparisons. The lack of observed differences in depression, anxiety, and parity in our sample is a strength of the study, but could also be the reason we do not see observed differences between groups. Previous studies of larger population level sample sizes have shown differences in mood distributions between cannabis exposure (81). In addition, participants did not use any other illicit substances and had relatively low exposure to tobacco. This is not representative of larger samples as co-substance use is very common, but our study design helped to minimize the impact of other drugs and substances on the brain. As our groups were well matched, the results suggest a potential weak difference that might be uniquely related to cannabis use, but not encompass these other factors. Our result provides the initial evidence for the potentially weak differences in parental brain responses to infant cues due to cannabis exposure. It would be important to replicate in larger samples and account for other variables that are correlated with cannabis use, such as depression and anxiety.

## Data availability statement

The datasets presented in this article are not readily available because they contain information that could compromise research participant consent and privacy. Due to the sensitivity of the human participant data and the potential for identification of de-identified participant, it would be important to make the data available to other researchers under a data-sharing agreement that provides for a commitment to data security approved by the corresponding author’s institution IRB. Requests to access the datasets should be directed to the corresponding author and the senior author (PK).

## Ethics statement

The studies involving humans were approved by University of Denver Institutional Review Board. The studies were conducted in accordance with the local legislation and institutional requirements. The participants provided their written informed consent to participate in this study.

## Author contributions

SP assisted in data collection, analyzed and interpreted the data, and drafted the manuscript. JM and AD assisted in data collection. XH and TY assisted in data analysis. TM contributed to the study design. PK conceived the study design, and assisted in data analysis. All authors contributed to the article and approved the submitted version.

## Funding

This work was supported by the National Institute of Health [R01HD090068; R21DA046556] and NARSAD Independent Investigator Grant.

## Conflict of interest

The authors declare that the research was conducted in the absence of any commercial or financial relationships that could be construed as a potential conflict of interest.

## Publisher’s note

All claims expressed in this article are solely those of the authors and do not necessarily represent those of their affiliated organizations, or those of the publisher, the editors and the reviewers. Any product that may be evaluated in this article, or claim that may be made by its manufacturer, is not guaranteed or endorsed by the publisher.

## References

[1] Anonymous. Committee Opinion No. 722: marijuana use during pregnancy and lactation. Obstet Gynecol (2017); 130:e205–e209; doi: 10.1097/AOG.0000000000002354, PMID: Retraction in: Obstet Gynecol. 2018 Jan;131(1):164-165 1873-233X. 28937574

[2] Cousijn J Goudriaan AE Ridderinkhof KR van den Brink W Veltman DJ Wiers RW. Approach-bias predicts development of cannabis problem severity in heavy cannabis users: results from a prospective FMRI study. PLoS One. (2012) 7:e42394. doi: 10.1371/journal.pone.0042394, PMID: 22957019 PMC3434213

[3] Mark K Desai A Terplan M. Marijuana use and pregnancy: prevalence, associated characteristics, and birth outcomes. Arch Womens Ment Health. (2016) 19:105–11. doi: 10.1007/s00737-015-0529-9, PMID: 25895138

[4] Anonymous. Monitoring health concerns related to marijuana in Colorado: 2020 report summary – Google drive. (2021) Available at: https://drive.google.com/drive/folders/1H9g7iwIIW-NMdDCgmtdgk8zvjwWcQGqd [Last accessed: 9/22/2022].

[5] Barba-Müller E Craddock S Carmona S Hoekzema E. Brain plasticity in pregnancy and the postpartum period: links to maternal caregiving and mental health. Arch Womens Ment Health. (2019) 22:289–99. doi: 10.1007/s00737-018-0889-z, PMID: 30008085 PMC6440938

[6] Hoekzema E Barba-Müller E Pozzobon C Picado M Lucco F García-García D. Pregnancy leads to long-lasting changes in human brain structure. Nat Neurosci. (2017) 20:287–96. doi: 10.1038/nn.4458, PMID: 27991897

[7] Kim P Capistrano CG Erhart A Gray-Schiff R Xu N. Socioeconomic disadvantage, neural responses to infant emotions, and emotional availability among first-time new mothers. Behav Brain Res. (2017) 325:188–96. doi: 10.1016/j.bbr.2017.02.001, PMID: 28163097 PMC5410181

[8] Kim P. Human maternal brain plasticity: adaptation to parenting. New Dir Child Adolesc Dev. (2016) 2016:47–58. doi: 10.1002/cad.20168 PMC5667351 27589497

[9] Laurent HK Ablow JC. A cry in the dark: depressed mothers show reduced neural activation to their own infant’s cry. Soc Cogn Affect Neurosci. (2012) 7:125–34. doi: 10.1093/scan/nsq091, PMID: 21208990 PMC3277361

[10] Kim P Leckman JF Mayes LC Feldman R Wang X Swain JE. The plasticity of human maternal brain: longitudinal changes in brain anatomy during the early postpartum period. Behav Neurosci. (2010) 124:695–700. doi: 10.1037/a0020884, PMID: 20939669 PMC4318549

[11] Swain JE. Baby stimuli and the parent brain: functional neuroimaging of the neural substrates of parent-infant attachment. Psychiatry Edgmont. (2008) 5:28–36. PMID: 19727273 PMC2695737

[12] Strathearn L Li J Fonagy P Montague PR. What’s in a smile? Maternal brain responses to infant facial cues. Pediatrics. (2008) 122:40–51. doi: 10.1542/peds.2007-1566, PMID: 18595985 PMC2597649

[13] Swain JE Lorberbaum JP Kose S Strathearn L. Brain basis of early parent–infant interactions: psychology, physiology, and in vivo functional neuroimaging studies. J Child Psychol Psychiatry. (2007) 48:262–87. doi: 10.1111/j.1469-7610.2007.01731.x, PMID: 17355399 PMC4318551

[14] Zhang K Rigo P Su X Wang M Chen Z Esposito G. Brain responses to emotional infant faces in new mothers and nulliparous women. Sci Rep. (2020) 10:9560. doi: 10.1038/s41598-020-66511-x, PMID: 32533113 PMC7293211

[15] Duarte-Guterman P Leuner B Galea LAM. The long and short term effects of motherhood on the brain. Front Neuroendocrinol. (2019) 53:100740. doi: 10.1016/j.yfrne.2019.02.004 30826374

[16] Barrière DA Ella A Szeremeta F Adriaensen H Même W Chaillou E. Brain orchestration of pregnancy and maternal behavior in mice: a longitudinal morphometric study. NeuroImage. (2021) 230:117776. doi: 10.1016/j.neuroimage.2021.117776, PMID: 33516895

[17] Barrett J Wonch KE Gonzalez A Ali N Steiner M Hall GB. Maternal affect and quality of parenting experiences are related to amygdala response to infant faces. Soc Neurosci. (2012) 7:252–68. doi: 10.1080/17470919.2011.609907, PMID: 21943083

[18] Hoekzema E Tamnes CK Berns P Barba-Müller E Pozzobon C Picado M. Becoming a mother entails anatomical changes in the ventral striatum of the human brain that facilitate its responsiveness to offspring cues. Psychoneuroendocrinology. (2020) 112:104507. doi: 10.1016/j.psyneuen.2019.104507, PMID: 31757430

[19] Brunton PJ Russell JA. Endocrine induced changes in brain function during pregnancy. Brain Res. (2010) 1364:198–215. doi: 10.1016/j.brainres.2010.09.062 20869351

[20] Brunton PJ Russell JA. The expectant brain: adapting for motherhood. Nat Rev Neurosci. (2008) 9:11–25. doi: 10.1038/nrn2280, PMID: 18073776

[21] Numan M. Parental behavior, reference module in neuroscience and Biobehavioral psychology. Netherlands: Elsevier (2017).

[22] Numan M. The parental brain: Mechanisms, development, and evolution. New York: Oxford University Press (2020).

[23] Russell JA Douglas AJ Ingram CD. Chapter 1 brain preparations for maternity — adaptive changes in Behavioral and neuroendocrine systems during pregnancy and lactation. An overview. In: Progress in brain research. The Maternal Brain Elsevier. (2001) 133:1–38. doi: 10.1016/S0079-6123(01)33002-9 11589124

[24] Witteman J van IJzendoorn MH Rilling JK Bos PA Schiller NO Bakermans-Kranenburg MJ. Towards a neural model of infant cry perception. Neurosci Biobehav Rev. (2019) 99:23–32. doi: 10.1016/j.neubiorev.2019.01.026, PMID: 30710581

[25] Bjertrup A Friis N Væver M Miskowiak K. Neurocognitive processing of infant stimuli in mothers and non-mothers: psychophysiological, cognitive and neuroimaging evidence. Soc Cogn Affect Neurosci. (2021) 16:428–38. doi: 10.1093/scan/nsab002, PMID: 33420780 PMC7990066

[26] Bjertrup AJ Friis NK Miskowiak KW. The maternal brain: neural responses to infants in mothers with and without mood disorder. Neurosci Biobehav Rev. (2019) 107:196–207. doi: 10.1016/j.neubiorev.2019.09.011, PMID: 31518637

[27] Rutherford HJV Yip SW Worhunsky PD Kim S Strathearn L Potenza MN. Differential responses to infant faces in relation to maternal substance use: an exploratory study. Drug Alcohol Depend. (2020) 207:107805. doi: 10.1016/j.drugalcdep.2019.107805, PMID: 31874448 PMC7060928

[28] Squire S Stein A. Functional MRI and parental responsiveness: a new avenue into parental psychopathology and early parent-child interactions? Br J Psychiatry. (2003) 183:481–3. doi: 10.1192/02-632, PMID: 14645017

[29] Nitschke JB Nelson EE Rusch BD Fox AS Oakes TR Davidson RJ. Orbitofrontal cortex tracks positive mood in mothers viewing pictures of their newborn infants. NeuroImage. (2004) 21:583–92. doi: 10.1016/j.neuroimage.2003.10.005 14980560

[30] Rigo P Kim P Esposito G Putnick DL Venuti P Bornstein MH. Specific maternal brain responses to their own child’s face: an fMRI meta-analysis. Dev Rev. (2019) 51:58–69. doi: 10.1016/j.dr.2018.12.001, PMID: 30872887 PMC6411077

[31] Strathearn L Kim S. Mothers’ amygdala response to positive or negative infant affect is modulated by personal relevance. Front Neurosci. (2013) 7:176. doi: 10.3389/fnins.2013.00176 24115918 PMC3792358

[32] Rigo P Esposito G Bornstein MH de Pisapia N Manzardo C Venuti P. Brain processes in mothers and nulliparous women in response to cry in different situational contexts: a default mode network study. Parenting. (2019) 19:69–85. doi: 10.1080/15295192.2019.1555430

[33] Grande LA Olsavsky AK Erhart A Dufford AJ Tribble R Phan KL. Postpartum stress and neural regulation of emotion among first-time mothers. Cogn Affect Behav Neurosci. (2021) 21:1066–82. doi: 10.3758/s13415-021-00914-9, PMID: 34128217 PMC8565500

[34] Moodie CA Suri G Goerlitz DS Mateen MA Sheppes G McRae K. The neural bases of cognitive emotion regulation: the roles of strategy and intensity. Cogn Affect Behav Neurosci. (2020) 20:387–407. doi: 10.3758/s13415-020-00775-8, PMID: 32133586

[35] Wan MW Downey D Strachan H Elliott R Williams SR Abel KM. The neural basis of maternal bonding. Avenanti A ed PLoS ONE. (2014) 9:e88436. doi: 10.1371/journal.pone.0088436, PMID: 24594508 PMC3942310

[36] Rutherford HJV Wallace NS Laurent HK Mayes LC. Emotion regulation in parenthood. Dev Rev. (2015) 36:1–14. doi: 10.1016/j.dr.2014.12.008, PMID: 26085709 PMC4465117

[37] Pilyoung K. How stress can influence brain adaptations to motherhood. Front Neuroendocrinol. (2021) 60:100875. doi: 10.1016/j.yfrne.2020.100875, PMID: 33038383 PMC7539902

[38] Crume TL Powers S Dufford AJ Kim P. Cannabis and pregnancy: factors associated with cannabis use among pregnant women and the consequences for offspring neurodevelopment and early postpartum parenting behavior. Curr Addict Rep. (2022) 9:195–202. doi: 10.1007/s40429-022-00419-6

[39] Gray TR Eiden RD Leonard KE Connors GJ Shisler S Huestis MA. Identifying prenatal cannabis exposure and effects of concurrent tobacco exposure on neonatal growth. Clin Chem. (2010) 56:1442–50. doi: 10.1373/clinchem.2010.147876, PMID: 20628142 PMC3163087

[40] Alshaarawy O Roskos SE Meghea CI. Tobacco cigarette and cannabis use among new mothers. Addict Abingdon Engl. (2021) 116:2572–6. doi: 10.1111/add.15372, PMID: 33314407 PMC8192585

[41] Odom GC Cottler LB Striley CW Lopez-Quintero C. Perceived risk of weekly cannabis use, past 30-Day cannabis use, and frequency of cannabis use among pregnant women in the United States. Int J Women's Health. (2020) 12:1075–88. doi: 10.2147/IJWH.S266540, PMID: 33235517 PMC7678496

[42] Volkow ND Han B Compton WM McCance-Katz EF. Self-reported medical and nonmedical cannabis use among pregnant women in the United States. JAMA. (2019) 322:167–9. doi: 10.1001/jama.2019.7982, PMID: 31211824 PMC6582258

[43] Chang JC Tarr JA Holland CL de Genna NM Richardson GA Rodriguez KL. Beliefs and attitudes regarding prenatal marijuana use: perspectives of pregnant women who report use. Drug Alcohol Depend. (2019) 196:14–20. doi: 10.1016/j.drugalcdep.2018.11.028, PMID: 30658220 PMC6756431

[44] Bayrampour H Zahradnik M Lisonkova S Janssen P. Women’s perspectives about cannabis use during pregnancy and the postpartum period: an integrative review. Prev Med. (2019) 119:17–23. doi: 10.1016/j.ypmed.2018.12.002, PMID: 30552948

[45] Taylor T Pack R Hilton G. “No one loves my baby more than me:” a qualitative study of patients’ decision-making regarding cannabis use in pregnancy. J Obstet Gynaecol Can. (2021) 43:672. doi: 10.1016/j.jogc.2021.02.078

[46] Crume TL Juhl AL Brooks-Russell A Hall KE Wymore E Borgelt LM. Cannabis use during the perinatal period in a state with legalized recreational and medical marijuana: the association between maternal characteristics, breastfeeding patterns, and neonatal outcomes. J Pediatr. (2018) 197:90–6. doi: 10.1016/j.jpeds.2018.02.005, PMID: 29605394

[47] Eiden RD Schuetze P Shisler S Huestis MA. Prenatal exposure to tobacco and cannabis: effects on autonomic and emotion regulation. Neurotoxicol Teratol. (2018) 68:47–56. doi: 10.1016/j.ntt.2018.04.007, PMID: 29727701 PMC6161361

[48] Hill M Sternberg A Suk HW Meier MH Chassin L. The intergenerational transmission of cannabis use: associations between parental history of cannabis use and cannabis use disorder, low positive parenting, and offspring cannabis use. Psychol Addict Behav. (2018) 32:93–103. doi: 10.1037/adb0000333, PMID: 29189023 PMC5805616

[49] Kim S Iyengar U Mayes LC Potenza MN Rutherford HJV Strathearn L. Mothers with substance addictions show reduced reward responses when viewing their own infant’s face. Hum Brain Mapp. (2017) 38:5421–39. doi: 10.1002/hbm.23731, PMID: 28746733 PMC5763911

[50] Landi N Montoya J Kober H Rutherford HJ Mencl WE Worhunsky PD. Maternal neural responses to infant cries and faces: relationships with substance use. Front Psych. (2011) 2:2. doi: 10.3389/fpsyt.2011.00032, PMID: 21720537 PMC3118477

[51] Lloyd-Fox S Blasi A Elwell CE. Illuminating the developing brain: the past, present and future of functional near infrared spectroscopy. Neurosci Biobehav Rev. (2010) 34:269–84. doi: 10.1016/j.neubiorev.2009.07.008 19632270

[52] Roos A Robertson F Lochner C Vythilingum B Stein DJ. Altered prefrontal cortical function during processing of fear-relevant stimuli in pregnancy. Behav Brain Res. (2011) 222:200–5. doi: 10.1016/j.bbr.2011.03.055, PMID: 21458497

[53] Wilcox T Biondi M. fNIRS in the developmental sciences. Wiley Interdiscip Rev Cogn Sci. (2015) 6:263–83. doi: 10.1002/wcs.1343, PMID: 26263229 PMC4979552

[54] Minagawa-Kawai Y Matsuoka S Dan I Naoi N Nakamura K Kojima S. Prefrontal activation associated with social attachment: facial-emotion recognition in mothers and infants: erratum. Cereb Cortex. (2009) 19:284–992. doi: 10.1093/cercor/bhn081 18515298

[55] Dudek J Colasante T Zuffianò A Haley DW. Changes in cortical sensitivity to infant facial cues from pregnancy to motherhood predict mother–infant bonding. Child Dev. (2020) 91:e198–217. doi: 10.1111/cdev.13182, PMID: 30511459

[56] Metz TD Borgelt LM. Marijuana use in pregnancy and while breastfeeding. Obstet Gynecol. (2018) 132:1198–210. doi: 10.1097/AOG.0000000000002878, PMID: 30234728 PMC6370295

[57] Robinson SM Sobell LC Sobell MB Leo GI. Reliability of the timeline Followback for cocaine, cannabis, and cigarette use. Psychol Addict Behav J Soc Psychol Addict Behav. (2014) 28:154–62. doi: 10.1037/a0030992, PMID: 23276315

[58] Sobell LC Maisto SA Sobell MB Cooper AM. Reliability of alcohol abusers’ self-reports of drinking behavior. Behav Res Ther. (1979) 17:157–60. doi: 10.1016/0005-7967(79)90025-1 426744

[59] Cox JL Holden JM Sagovsky R. Detection of postnatal depression. Development of the 10-item Edinburgh postnatal depression scale. Br J Psychiatry J Ment Sci. (1987) 150:782–6. doi: 10.1192/bjp.150.6.782, PMID: 3651732

[60] Shrestha SD Pradhan R Tran TD Gualano RC Fisher JRW. Reliability and validity of the Edinburgh postnatal depression scale (EPDS) for detecting perinatal common mental disorders (PCMDs) among women in low-and lower-middle-income countries: a systematic review. BMC Pregnancy Childbirth. (2016) 16:72. doi: 10.1186/s12884-016-0859-2, PMID: 27044437 PMC4820998

[61] Bunevicius A Kusminskas L Bunevicius R. P02-206 validity of the Edinburgh postnatal depression scale. Eur Psychiatry. (2009) 24:1. doi: 10.1016/S0924-9338(09)71129-0 19667749

[62] Pinquart M Sörensen S. Associations of stressors and uplifts of caregiving with caregiver burden and depressive mood: a meta-analysis. J Gerontol Ser B. (2003) 58:P112–28. doi: 10.1093/geronb/58.2.P112 12646594

[63] Radloff Lenore. The CES-D scale: a self-report depression scale for research in the general population – Lenore sawyer Radloff, 1977. (1977). Available at: [Last accessed: 9/27/2022https://journals.sagepub.com/doi/abs/10.1177/014662167700100306].

[64] Lewinsohn PM Seeley JR Roberts RE Allen NB. Center for Epidemiologic Studies Depression Scale (CES-D) as a screening instrument for depression among community-residing older adults. Psychol Aging. (1997) 12:277–87. doi: 10.1037/0882-7974.12.2.277, PMID: 9189988

[65] Spielberger C. State-trait anxiety inventory: Bibliography. 2nd ed. Palo Alto: Consulting Psychologists Press (1989).

[66] Spielberger CD Gonzalez-Reigosa F Martinez-Urrutia A Natalicio LF Natalicio DS. Manual for the state-trait anxiety inventory. Revista Interamericana De Psicología/Interamerican Journal of Psychology, Consulting Psychologists Press (1983).

[67] Morais Guilherme Augusto Zimeo Balardin JB Sato JR. FNIRS Optodes’ location decider (FOLD): a toolbox for probe arrangement guided by brain regions-of-interest | scientific reports. (2018). Available at: https://www.nature.com/articles/s41598-018-21716-z [Last accessed: 9/27/2022]. 10.1038/s41598-018-21716-z PMC5820343 29463928

[68] Ozawa S Kanayama N Hiraki K. Emotion-related cerebral blood flow changes in the ventral medial prefrontal cortex: an NIRS study. Brain Cogn. (2019) 134:21–8. doi: 10.1016/j.bandc.2019.05.001, PMID: 31102883

[69] Anonymous. Cool Edit Pro Version 2.0, Syntrillium Software, Phoenix, AZ. (2002).

[70] Kim P Tribble R Olsavsky AK Dufford AJ Erhart A Hansen M. Associations between stress exposure and new mothers’ brain responses to infant cry sounds. NeuroImage. (2020) 223:117360. doi: 10.1016/j.neuroimage.2020.117360, PMID: 32927083 PMC8291268

[71] Schneider W Eschman A Zuccolotto A. (2002). E-Prime Reference Guide. Pittsburgh, PA: Psychology Software Tools.

[72] Huppert TJ Hoge R Dale AM Franceschini MA Boas DA. Quantitative spatial comparison of diffuse optical imaging with blood oxygen level-dependent and arterial spin labeling-based functional magnetic resonance imaging. J Biomed Opt. (2006) 11:064018. doi: 10.1117/1.2400910, PMID: 17212541 PMC2670188

[73] Grossmann T Johnson MH. Selective prefrontal cortex responses to joint attention in early infancy. Biol Lett. (2010) 6:540–3. doi: 10.1098/rsbl.2009.1069, PMID: 20106861 PMC2936215

[74] Anonymous. MATLAB. MATLAB version: 9.2.0.556344 (R2017a), Natick, Massachusetts: The MathWorks Inc.; 2022. (2018).

[75] Santosa H Zhai X Fishburn F Huppert T. The NIRS brain AnalyzIR toolbox. Algorithms. (2018) 11:73. doi: 10.3390/a11050073 PMC11218834 38957522

[76] Anonymous. IBM SPSS statistics for windows. IBM Corp. Released 2021. IBM SPSS Statistics for Windows, Version 28.0. Armonk, NY: IBM Corp (2021).

[77] Manelis A Huppert T Rodgers E Swartz HA Phillips ML. The role of the right prefrontal cortex in recognition of facial emotional expressions in depressed individuals: fNIRS study. J Affect Disord. (2019) 258:151–8. doi: 10.1016/j.jad.2019.08.006, PMID: 31404763 PMC6710146

[78] Balconi M Kopis N Angioletti L. Does aesthetic judgment on face attractiveness affect neural correlates of empathy for pain? A fNIRS study Exp Brain Res. (2020) 238:2067–76. doi: 10.1007/s00221-020-05867-y, PMID: 32638037

[79] Fishburn F Norr M Medvedev A Vaidya CJ. Sensitivity of fNIRS to cognitive state and load. Front Hum Neurosci. (2014) 8:76. doi: 10.3389/fnhum.2014.00076, PMID: 24600374 PMC3930096

[80] Swain JE Kim P Spicer J Ho SS Dayton CJ Elmadih A. Approaching the biology of human parental attachment: brain imaging, oxytocin and coordinated assessments of mothers and fathers. Brain Res. (2014) 1580:78–101. doi: 10.1016/j.brainres.2014.03.007, PMID: 24637261 PMC4157077

[81] Goodwin RD Zhu J Heisler Z Metz TD Wyka K Wu M. Cannabis use during pregnancy in the United States: the role of depression. Drug Alcohol Depend. (2020) 210:107881. doi: 10.1016/j.drugalcdep.2020.107881, PMID: 32143978

