# Supplementary material for: Cannabis use during pregnancy and hemodynamic responses to infant cues in pregnancy: an exploratory study
Source: Front Psychiatry. 2023 Sep 6;14:1180947. doi: 10.3389/fpsyt.2023.1180947 (PMC10512021; doi:10.3389/fpsyt.2023.1180947)
Supplement: Supplementary file 1 [file Table_1.DOCX]

Supplementary

*fNIRS deoxygenated analysis of the association between cannabis use and brain activation to infant cry and infant picture*

The two-way interaction between sound (infant cry and matched white noise) x group (cannabis using and non-cannabis using) covarying for income was significant across 2 deoxygenated channels in the left dorsolateral PFC (S2:D1) and one in the middle dorsomedial PFC (S4:D4). In the dorsomedial PFC channel, the cannabis group exhibited the expected opposite pattern to oxygenated hemoglobin (cannabis infant cry µβ= -4.05+/- 12.61, control infant cry µβ= .10+/- 15.32, cannabis white noise µβ= 2.74 +/- 8.98, control white noise µβ= -.12 +/- 9.61; F(1) = 5.28, p = .025). For the dorsolateral channel, we saw a similar expected pattern (cannabis infant cry µβ= -1.00+/- 7.07, control infant cry µβ= -.10 +/- 3.88, cannabis white noise µβ= 2.12 +/- 8.10 control white noise µβ= .06 +/- 3.26; F(1) = 4.66, p = .035). No results survived multiple correction with the Benjamini-Hochberg test. There were no significant deoxygenated channels for infant picture.
