# Supplementary figures and images for: Cannabis use during pregnancy and hemodynamic responses to infant cues in pregnancy: an exploratory study
Source: Front Psychiatry. 2023 Sep 6;14:1180947. doi: 10.3389/fpsyt.2023.1180947 (PMC10512021; doi:10.3389/fpsyt.2023.1180947)

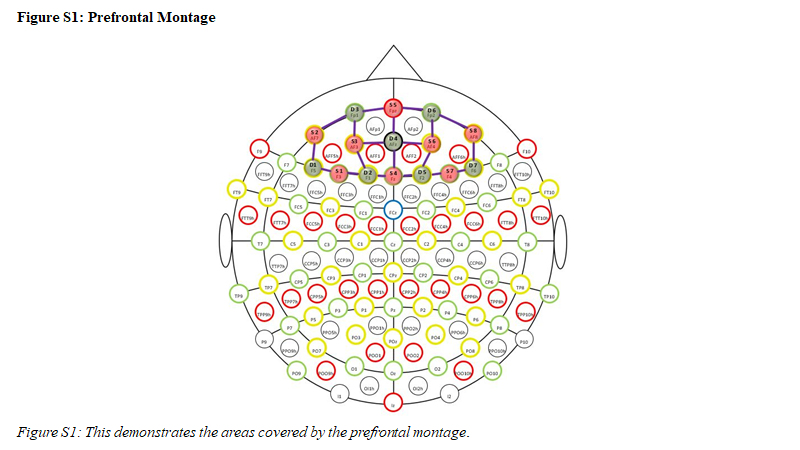

Supplement: Supplementary file 2 [file Image_1.TIF]
